# Evaluation of Two Different Treatments for Larch Logs as Substrates to Cultivate *Ganoderma tsugae* in the Forest

**DOI:** 10.3390/life15010039

**Published:** 2024-12-31

**Authors:** Lei Xia, Xiao Tan, Peng Wang, Dahai Yang, Yang Zhang, Yanru Cui, Ya Yu, Weidong Zhang, Xiao Huang, Jiawei Wen

**Affiliations:** Jilin Academy of Agricultural Sciences, Northeast Agricultural Research Center of China, Changchun 130033, China; fushun1020@yeah.net (L.X.); wsymin@163.com (X.T.); wangpengtongbai@163.com (P.W.); darkangel1999@163.com (D.Y.); 2253677@163.com (Y.Z.); nkycyr@126.com (Y.C.); yuya0713@163.com (Y.Y.); baiyang5447@163.com (W.Z.); huangxiaoxiwang@sina.com (X.H.)

**Keywords:** *Ganoderma tsugae*, larch logs, yield, agronomic traits, nutritional value

## Abstract

Larch wood, a prevalent cultivation medium for *Ganoderma tsugae*, has yet to be scrutinized concerning the differential impacts of sterilized and non-sterilized substrates on the growth and development of this fungus. Our present investigation sought to elucidate these effects in a forest-like environment. After larch wood segments were sun-dried, they were divided into two groups; one group was bagged and autoclaved, while the other group was bagged without any treatment. Subsequently, all segments were inoculated with the *G. tsugae* strain HLXL1 and ensconced under the canopy of a *Pinus koraiensis* forest, thereby approximating the conditions of natural growth. Wild *G. tsugae* was used as the control. Data on agronomic traits, production days, fruiting body yield, and effective constituent content were analyzed. The results indicated no significant differences between sterilized and non-sterilized substrates in terms of agronomic traits. However, the mineral content and bioactive compounds in *G. tsugae* fruiting bodies significantly differed across various growth stages. The outcomes were optimal for non-sterilized substrates, followed by sterilized substrates, while the wild strains were markedly less effective than the cultivated ones.

## 1. Introduction

*Ganoderma tsugae* Murrill (*G. tsugae*), which is commonly known as hemlock reishi, is a Ganoderma fungus classified in the family Ganodermataceae of the basidiomycete class. Wild *Ganoderma* spp. mainly grows in tropical and subtropical climates and temperate regions, including China, Japan, Thailand, Vietnam, and America [1,2,3]. In China, it mainly grows in the Changbai Mountain area. *Ganoderma* has been recognized and used extensively as a traditional medicine to improve health and prolong life in East Asia for more than 2000 years [4,5,6]. In the past 20–30 decades or so, increasing awareness of the potential health benefits of Ganoderma species has led to production of this medicinal mushroom in the United States and Canada. The latest available estimates put the annual value of *G. lucidum* products worldwide at more than USD 1.6 billion [7,8]. Due to its rarity in nature and limited availability, the natural collection of this medicinal mushroom remains insufficient for global commercial use. The increasing global demand for *G. tsugae*, coupled with its limited natural availability, has necessitated the development of commercial artificial cultivation methods.

*Ganoderma* was initially successfully cultivated in 1 m long natural cut logs without sterilization in China in 1969 [9]. This required a long incubation period (2–3 years) to harvest mature fruiting bodies [10]. Since then, various techniques for *G. tsugae* cultivation have been developed. Among these, both the wood-log and substitute bag culture techniques have been widely employed for the production of *G. lucidum* [10,11,12]. Wood-log cultivation includes sterilized short wood logs, unsterilized wood logs, and stump cultivation. Among these, short wood-log cultivation holds obvious advantages, including the shortest growth cycle and higher yield. At present, short wood-log cultivation methods have been favored by the majority of cultivators [11]. *G. tsugae*-inoculated wood logs are transferred to a natural or artificial forest environment, or a mushroom house, in order to achieve two harvests within a 2-year cultivation period [13,14].

*G. tsugae* contains several components responsible for its bioactive and pharmacological properties [15,16]. Polysaccharides and triterpenoids are the major bioactive constituents of *Ganoderma* [17]. Furthermore, the active compounds in *G. tsugae* and other medicinal species of the genus have exhibited wide-ranging effects, including anti-cancer and anti-tumor activity (by increasing TNF-a, IFN-g, and IL-2), anti-HIV activity (by inhibiting virus proliferation), and anti-aging activity (by increasing a-DNA polymerase) [17,18,19,20,21]. Various polysaccharides have demonstrated anticancer, antitumor, antiviral, and antioxidant properties, along with the ability to stimulate the immune system [22,23,24]. In addition, triterpenoids have exhibited cytotoxic effects on cancer cells, and are recognized as potential anticancer agents. In actual production, the quantity of the bioactive constituents vary among strains, due to the origins, cultivation techniques, and growing conditions. In addition, bioactive components are the most important considered factors in the market value of medicinal mushrooms. In order to increase the content of active ingredients and yield, it is necessary to optimize the substrate and cultivation conditions.

The yield and bioactive components of *G. tsugae* on short wood logs were significantly affected by different growth conditions [13,14]. Thus, developing and optimizing detailed research on *G. tsugae* substrates is crucial to enhance practical production. The study evaluated the impact of these substrates on the bioactive components present in the fruiting bodies of *G. tsugae* and yield. These study findings would offer valuable insights into the suitability of variously treated larch logs for the growth, quantity, and quality of *G. tsugae*. Furthermore, this information would serve as a valuable resource for *G. tsugae* growers, aiding them in enhancing production levels and income.

## 2. Materials and Methods

### 2.1. Inoculum Source and Spawn Preparation

The *G. tsugae* strain HLXL1 used for the present experiment was aseptically isolated and purified from wild fruiting bodies of *G. tsugae* in Jilin Province, China (east longitude 128°49′, north latitude 42°26′). The mycelia were cultured on potato dextrose agar (PDA), and incubated in the dark at 25 °C until the plate surfaces were colonized by mycelia.

### 2.2. Substrate Preparation

Trees (*Larix gmelinii* [Rupr.] Kuzen. [*L. gmelinii*]) with a diameter of 18–20 cm were cut down to 20–25 cm in length and lightly air-dried for 15–20 days in a clean and well-ventilated place in order to obtain a relative moisture content of 35–42% [14]. Then, 10 short logs were tightly bundled and placed in a polypropylene bag. The short logs of *L. gmelinii* (for 600 bags) were sterilized (Y1 treatment) in normal air pressure for nine hours at 100 °C. After sterilization, the sterilized bags were allowed to cool in a chamber for one day, while another 600 bags of short *L. gmelinii* logs were unsterilized by direct inoculation (Y2 treatment). The strains that grew in the rotten wood of wild larch trees was used as the control (CK).

### 2.3. Inoculum Preparation

A PDA suitable for mother-culture media was cultured for 20 days to prepare the planting spawn. The cultivated spawn substrate was prepared using 80% sawdust, 15% wheat bran, 1.5% sucrose, 2% soybean meal, and 1.5% calcium oxide. This was maintained at 55–60% moisture content, and autoclaved for one hour at 121 °C. After cooling, the pure culture of *G. tsugae* strain HLXL1 was inoculated into sterilized bags to avoid any possibility of contamination. Then, these inoculated bags were incubated in the dark at 20–25 °C with 60–70% relative humidity for 45–50 days, until the entire package that contained the substrate turned white due to fungal mycelial proliferation.

### 2.4. Inoculation for Wood Logs

The *G. tsugae* planting spawn was aseptically inoculated on the prepared short logs at the rate of 300 g/bag. Then, the bags were sealed to avoid contamination and incubated in well-ventilated and sterilized dark conditions for spawn growth at 22–25 °C with 70–80% relative humidity for 5–6 months, until the mycelium fully colonized in the larch logs. All bags were transferred to the cultivation base in Changbai Shanzhiyuan Ecological Agriculture Co., Changbai County, Jilin Province, China (127°56′30″–128°17′34″ E, 41°58′03″–41°21′37″ N). Next, after being removed from the polypropylene bag, the short logs were buried at half their depth in a red pine forest, with a row spacing of 20–30 cm and a distance of 15 cm between each other, for the randomized treatment group. Then, these were covered with a thin layer of pine needles to maintain proper soil moisture. The temperature was regularly recorded throughout the season. The relative humidity was controlled by the artificial spray system at 60–70% during primordia formation and at 75–85% during fruiting body formation.

### 2.5. Assay for Growth Performance and Yield of G. tsugae

The following growth parameters were recorded: growth cycle, pileus diameter (mm) and thickness (mm), stipe length (mm) and diameter (mm), fresh weight (g) per fruiting body, and fresh weight (g)/100 fruiting bodies. Furthermore, the morphological characteristics of the fruiting bodies at maturity, such as color and shape, were observed.

### 2.6. Analysis of Bioactive and Mineral Compounds

The *G. tsugae* primordia, mature fruiting bodies, and spore released fruiting bodies were sampled. The concentrations of triterpenes; polysaccharides; total protein, fat, and fiber; and trace elements were tested in duplicate. All test analyses were performed by the Qingdao Standard Testing Group (Qingdao, China).

### 2.7. Statistical Analysis

The original data were analyzed using Microsoft Excel (Redmond, WA, USA). The differences between group means were evaluated using Duncan’s multiple range test at a 95% confidence level (*p* < 0.05). The statistical and correlation analyses were conducted using SPSS 19.0 (IBM, Inc., Armonk, NY, USA).

## 3. Results

### 3.1. Morphology and Fruiting Characteristics of G. tsugae

Table 1 presents the agronomic characteristics of two differently treated wood substrates and CK regarding the growth performance of *G. tsugae*. The growth performance and yield of *G. tsugae* cultivated artificially were unaffected. The weights of 100 fruiting bodies and individual fruiting bodies from Y1, Y2, and CK showed no significant differences. The diameter and thickness of the pileus of Y1 were significantly greater, at 135.93 mm and 27.43 mm, respectively, compared to 106.83 mm and 23.57 mm for CK. However, Y2 mushrooms’ pileus diameter and thickness were not significantly different from those of Y1 and CK.

As shown in Table 2, throughout the entire fertility cycle, the order was CK > Y2 > Y1. Y1 had the shortest developmental cycle. The growth cycle was longer for CK. There was no significant difference in average pollution rate between Y1 and Y2.

**Figure 1:**
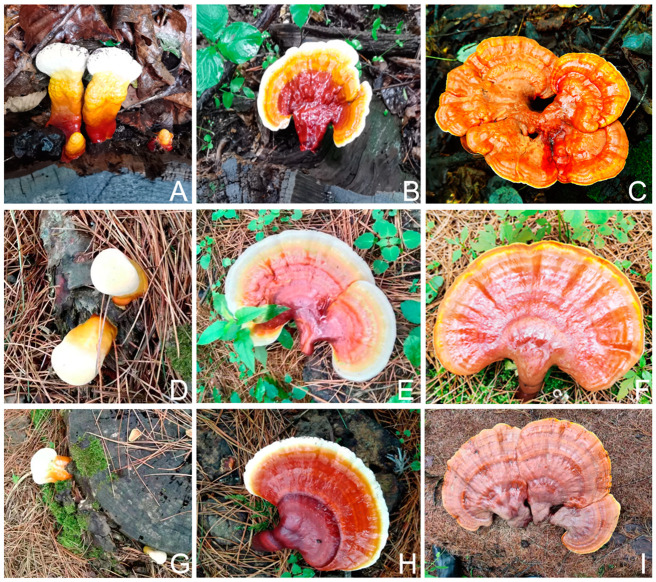
Morphological characteristics of the fruiting bodies of *G. tsugae* in three different growth stages grown on different substrates. (**A**), primordium of CK; (**B**), immature fruiting body of CK; (**C**), mature fruiting body of CK. (**D**), primordium of Y1; (**E**), immature fruiting body of Y1; (**F**), mature fruiting body of Y1. (**G**), primordium of Y2; (**H**) immature fruiting body of Y2; (**I**), mature fruiting body of Y2.

As shown in Figure 1, there was no significant difference in the morphological characteristics of *G. tsugae* in the three different growth stages on different substrates. The fruiting body at maturity was reniform or fan-shaped and reddish brown. The wild strain presented with a slightly lighter color, and was combinatorial in shape. Furthermore, the mature fruiting body in CK was smaller, when compared to that in Y1 and Y2, which is consistent with the statistical analysis results (Table 1).

### 3.2. Bioactive Contents of G. tsugae

The fruiting bodies of *G. tsugae* were sampled from Y1, Y2, and CK at different growth stages for bioactive component analysis. The bioactive components of *G. tsugae* at each growth stage varied significantly across different substrates, and those from Y2 had obvious advantages. Through data analysis, the contents of triterpenes and polysaccharides were obviously different. The triterpene and fat contents were higher in Y2, whereas Y1 had the highest levels of polysaccharides and proteins. CK had the lowest content in all categories (Table 3). In the mature fruiting bodies of *G. tsugae*, the bioactive components in Y2 excluding fiber, had significantly higher contents compared to Y1, whereas those in CK did not differ significantly (Table 4). In post-spore-release fruiting bodies of *G. tsugae*, the main active components of triterpenoids and polysaccharides were significantly higher in Y2 compared to the others, whereas those in Y1 were markedly lower (Table 5).

### 3.3. Mineral Contents of G. tsugae

The analysis of important minerals in the fruiting bodies of *G. tsugae* is detailed for all samples. The mineral contents in every growth stage of *G. tsugae* cultured on different substrates significantly varied. As shown in Table 6, the contents of Ca, Fe, Cu, Zn, and Se in the primordium of *G. tsugae* in Y1 were about twice as abundant as Y2, while these levels were the lowest in CK. In the mature fruiting bodies of *G. tsugae*, levels of iron (Fe), copper (Cu), zinc (Zn), and selenium (Se) with the exception of calcium (Ca) in CK were significantly higher (Table 7), while the calcium content in Y1 was considerably greater compared to the other samples. Table 8 shows that the levels of Fe, Cu, and Se were highest in CK, while Y1 significantly enhanced the levels of Ca and Zn. However, Y2 presented lower levels of all minerals throughout the growth process of *G. tsugae*.

### 3.4. The Result of Scatter Plot

In the present study, three types of larch (sterilization, unsterilized, and wild rotting wood) were used as the substrates. There were no significant differences in cap diameter or thickness in the treatment groups. The morphological characteristics of *G. tsugae* fruiting bodies grown on the three substrates were similar for all samples, and the weight was relatively stable.

**Figure 2:**
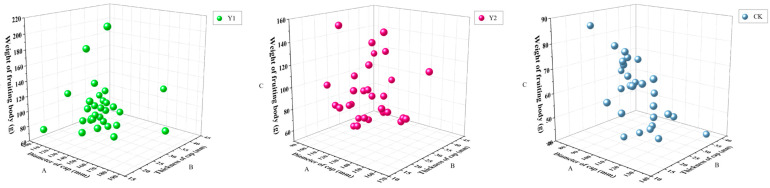
Scatter plot of the agronomic characteristics of fruiting bodies. A (mm): Diameter of the cap, B (mm): Cap thickness, C (g): Per Fruiting body Weight.

As shown in Figure 2, The scatter plot for the agronomic characteristics of fruiting bodies revealed that mushrooms grown from Y1 were more homogeneous, while those grown from Y2 and CK were more dispersed, but the difference was not significant. Uniformly sized mushrooms are better suited for uniform collection, thereby reducing the cost of labor. Weight, shape, and color are a good combination of criteria to use when evaluating mushrooms.

## 4. Discussion

A number of studies have revealed that triterpenoids and polysaccharides are the most important active components of *G. tsugae*. Various factors affect the content of triterpenoids and polysaccharides in mushrooms, such as the type of substrate, its physical and biochemical properties, the amount of nutrients added in the substrates, and the types of nutrients [25]. In China, *G. tsugae* produces little spore, so it is harvested before the spores are released. In the present study, the triterpenes and polysaccharides content were the highest in the whole growth process of *G. tsugae* with Y2 as the substrate. The triterpenes and polysaccharides content were 2.06% and 0.55%, respectively, for Y1, 2.30% and 0.77%, respectively, for Y2, and 2.30% and 0.70%, respectively, for CK in mature fruiting bodies. According to the results of this study, the Y2 substrate had the potential to serve as an excellent umnt factor for unsterilized larches (Y2) as desirable substrates to cultivate *G. tsugae* mushrooms, when compared to sterilized larches (Y1), was that these lead to more cost savings. In the Chinese market, the quality determines the price of *G. tsugae*. Thus, Y2 is an excellent medium to cultivate *G. tsugae* for commercial use.

The major ingredients in the substrates are the key factors that affect the mineral spectrum of the fruiting bodies of edible fungi. There are significant differences in the uptake of individual elements, and these elements can be found to occur naturally in substrate materials. Among the minerals in the present study, Ca had the highest level in the fruiting bodies, followed by Fe, Zn, Se, and Cu. Furthermore, several mineral contents in CK were the highest. Zn and Se, as accumulated metals, have a significant effect on the function of humans [26,27]. The fruiting bodies exhibited elevated Zn concentrations when grown on the Y1 substrate, while the Y2 cultivation substrate is suitable for producing mushrooms with high Se content.

The fruiting body formation of *G. tsugae* can be divided into three stages: the primordium stage, the fruiting maturity stage, and the sporulation stage. When the primordium is formed, this should be distanced to allow the primordium to grow, facilitating the better growth of the fruiting body. The present study results revealed that no matter what kind of substrate, there was no significant difference in active components before and after the spore was released. The reason may be due to the low sporulation, which did not affect the content of effective substances. There was no obvious difference between the artificial cultivation and wild growth of *G. tsugae*. In the three kinds of substrates, the Ca and Fe content was significantly higher before the spores were released, when compared to that after the spores were released. This shows that spore release has great influence on the content of trace elements. These results reveal that growers do not need to worry of the effects of spore release on the quality of *G. tsugae* and late harvests.

*G. tsugae* is best grown in the forest to achieve the highest efficacy. The main advantages of growing *G. tsugae* under the forest are that this provides value to private forest owners, and creates additional value for the maintenance of deadwood and retention trees, which has a positive impact on biodiversity, and increases carbon storage in forests. However, the production of *G. tsugae* under the forest to imitate wild growth is seasonal, time-consuming, and difficult to control. Furthermore, the fruiting in-season is weather-dependent, and competitive with the occurrence of the fungi [28]. The most common fungal contaminant is *Penicillium* sp., which infects wood segments or fruiting bodies, and adverse weather conditions such as prolonged drought or high temperatures can impede the development of fruiting bodies, leading to reduced yields, inferior quality, and economic losses for growers. Several factors, including the substrate nutrients and physical structure, growth parameters, and environment (outdoor vs. indoor cultivation), affect the growth performance, yield, and bioactive components of *Ganoderma* sp. [13,14,29,30]. The present study investigated the effects of sterilized and unsterilized wood log treatments on the probability of successful fruiting, yield, and bioactive components of *G. tsugae*, understory cultivation. The non-autoclave treatment of larch wood had stronger effects on the nutritional and mineral components of fruiting bodies. *G. tsugae* strains differ in its ability to decompose wood, such as degraded lignin and absorbed nutrition resulting in different growth responses to the logs in the forest [31]. Thus, further studies are warranted to deepen our understanding of the optimal substrate of *G. tsugae* strains for understory mushroom cultivation. Identifying the most effective cultivation approach for *G. tsugae* strains with several types of logs can enhance the conversion efficiency of low-value lignocellulosic biomass into high-value crops.

## 5. Conclusions

The present study confirms the advantage of Y2 as a substrate to cultivate *G. tsugae*. This substrate was proven to be favorable to provide an equal yield of mushrooms, along with high contents of triterpenes and polysaccharides, and minerals such as Cu, Fe, Zn Se that can be obtained in mature fruiting bodies. Therefore, Y2 can be used in the commercial cultivation of *G. tsugae* which can produce high quality *G. tsugae*. These results provide a practical method for mushroom growers.

## Figures and Tables

**Table 1:** Agronomic traits of *G. tsugae* cultivated on differently treated wood substrates (mean ± SD).

Substrate	Weight per Hundred Fruiting Bodies (g)	Fruiting Body Yield (g)	Diameter of Pileus (mm)	Thickness of Pileus (mm)	Diameter of Stipe (mm)	Length of Stipe (mm)
Y1	11,781.18 ± 1046.53 a	176.10 ± 27.89 a	135.93 ± 2.14 a	27.43 ± 1.31 a	27.20 ± 2.17 a	77.47 ± 1.0 a
Y2	11,456.28 ± 2662.98 ab	159.57 ± 5.50 a	122.83 ± 3.73 ab	26.33 ± 1.98 ab	24.63 ± 0.81 ab	52.73 ± 5.51 b
CK	nd	158.67 ± 28.09 a	106.83 ± 13.80 b	23.57 ± 1.68 b	23.70 ± 1.31 b	75.63 ± 2.15 a

Note: Different lowercase letters denote significant differences in each column (*p* < 0.05). CK: *G. tsugae* grows in the wild on rotting larch wood, Y1: *G. tsugae* grows on sterilized larch, Y2: *G. tsugae* grows on unsterilized larch. nd: not detected.

**Table 2:** Growth cycle and contamination rate of *G. tsugae*.

Substrate	Primordial Occurrence Time (d)	Growth Cycle (d)	Inoculation Contamination Rate (%)
Y1	27	62	2.3%
Y2	32	65	3.1%
CK	41	83	nd

Note: CK: *G. tsugae* grows in the wild on rotting larch wood, Y1: *G. tsugae* grows on sterilized larch, Y2: *G. tsugae* grows on unsterilized larch. nd: not detected.

**Table 3:** Nutritional value of primordium of *G. tsugae* when cultivated on different substrates (g 100 g^−1^).

Substrate	Triterpene	Polysaccharose	Protein	Fat	Fiber
Y1	2.50 ± 0.00 c	0.96 ± 0.04 a	27.65 ± 0.35 a	2.50 ± 0.14 b	11.60 ± 0.00 b
Y2	4.96 ± 0.01 a	0.73 ± 0.01 b	19.80 ± 0.71 b	2.90 ± 0.14 a	19.55 ± 0.07 a
CK	3.17 ± 0.00 b	0.56 ± 0.03 c	11.90 ± 0.00 c	3.05 ± 0.07 a	4.10 ± 0.00 c

Note: Different lowercase letters denote significant differences in each column (*p* < 0.05). CK: *G. tsugae* grows in the wild on rotting larch wood, Y1: *G. tsugae* grows on sterilized larch, Y2: *G. tsugae* grows on unsterilized larch.

**Table 4:** Nutritional value of mature fruiting bodies of *G. tsugae* when cultivated on different treated wood substrates (g 100 g^−1^).

Substrate	Triterpene	Polysaccharose	Protein	Fat	Fiber
Y1	2.06 ± 0.00 b	0.55 ± 0.06 b	12.20 ± 0.14 c	1.75 ± 0.07 b	27.95 ± 0.07 a
Y2	2.30 ± 0.00 a	0.77 ± 0.00 a	15.80 ± 0.42 b	2.40 ± 0.00 a	20.35 ± 0.07 c
CK	2.30 ± 0.00 a	0.70 ± 0.03 a	25.6 ± 0.14 a	2.60 ± 0.14 a	24.25 ± 0.07 b

Note: Different lowercase letters denote significant differences in each column (*p* < 0.05). CK: *G. tsugae* grows in the wild on rotting larch wood, Y1: *G. tsugae* grows on sterilized larch, Y2: *G. tsugae* grows on unsterilized larch.

**Table 5:** Nutritional value of post-spore-release fruiting bodies of *G. tsugae* when cultivated on different substrates (g 100 g^−1^).

Substrate	Triterpene	Polysaccharose	Protein	Fat	Fiber
Y1	2.76 ± 0.00 b	0.76 ± 0.02 ab	8.17 ± 0.02 b	1.85 ± 0.07 b	27.05 ± 0.07 a
Y2	3.27 ± 0.00 a	0.85 ± 0.06 a	5.23 ± 0.18 c	1.75 ± 0.07 b	7.75 ± 0.07 c
CK	2.59 ± 0.00 c	0.66 ± 0.04 b	11.95 ± 0.07 a	3.30 ± 0.28 a	26.55 ± 0.07 b

Note: Different lowercase letters denote significant differences in each column (*p* < 0.05). CK: *G. tsugae* grows in the wild on rotting larch wood, Y1: *G. tsugae* grows on sterilized larch, Y2: *G. tsugae* grows on unsterilized larch.

**Table 6:** The contents of trace elements in primordium of *G. tsugae* grown on different treated wood substrates.

Substrate	Ca (mg kg^−1^)	Fe (mg kg^−1^)	Cu (mg kg^−1^)	Zn (mg kg^−1^)	Se (µg kg^−1^)
Y1	0.18 ± 0.00 a	385.5 ± 0.71 a	28.85 ± 0.35 a	95.20 ± 0.00 a	222.50 ± 3.54 a
Y2	908.50 ± 7.781 b	159.50 ± 2.12 b	14.35 ± 0.49 c	55.05 ± 0.78 c	85.25 ± 0.21 b
CK	797.00 ± 2.83 c	78.3 ± 0.42 c	16.75 ± 0.07 b	59.50 ± 0.14 b	63.65 ± 1.48 c

Note: Different lowercase letters denote significant differences in each column (*p* < 0.05). CK: *G. tsugae* grows in the wild on rotting larch wood, Y1: *G. tsugae* grows on sterilized larch, Y2: *G. tsugae* grows on unsterilized larch.

**Table 7:** The contents of trace elements in mature fruiting bodies of *G. tsugae* grown on different treated wood substrates.

Substrate	Ca (mg kg^−1^)	Fe (mg kg^−1^)	Cu (mg kg^−1^)	Zn (mg kg^−1^)	Se (µg kg^−1^)
Y1	1220.00 ± 14.14 a	52.05 ± 1.20 b	11.55 ± 0.07 b	49.75 ± 0.35 b	29.30 ± 0.14 c
Y2	861.50 ± 7.78 b	52.15 ± 0.92 b	11.50 ± 0.00 b	35.80 ± 0.28 c	58.55 ± 0.64 b
CK	805.50 ± 2.12 c	97.00 ± 0.57 a	20.65 ± 0.07 a	65.15 ± 0.35 a	63.20 ± 0.99 a

Note: Different lowercase letters denote significant differences in each column (*p* < 0.05). CK: *G. tsugae* grows in the wild on rotting larch wood, Y1: *G. tsugae* grows on sterilized larch, Y2: *G. tsugae* grows on unsterilized larch.

**Table 8:** The contents of trace elements in post-spore-release fruiting bodies of *G. tsugae* grown on different treated wood substrates.

Substrate	Ca (mg kg^−1^)	Fe (mg kg^−1^)	Cu (mg kg^−1^)	Zn (mg kg^−1^)	Se (µg kg^−1^)
Y1	933.00 ± 1.41 a	53.35 ± 0.78 b	13.90 ± 0.14 b	76.05 ± 0.49 a	29.40 ± 0.28 c
Y2	573.50 ± 2.12 c	47.35 ± 0.35 c	10.55 ± 0.49 c	27.25 ± 0.49 c	41.65 ± 0.35 b
CK	654.50 ± 0.71 b	62.60 ± 0.42 a	17.60 ± 0.14 a	52.10 ± 0.28 b	50.45 ± 0.35 a

Note: Different lowercase letters denote significant differences in each column (*p* < 0.05). CK: *G. tsugae* grows in the wild on rotting larch wood, Y1: *G. tsugae* grows on sterilized larch, Y2: *G. tsugae* grows on unsterilized larch.

## Data Availability

The raw data supporting the conclusions of this article will be made available by the authors on request.
